# H2A.X promotes endosperm-specific DNA methylation in Arabidopsis thaliana

**DOI:** 10.21203/rs.3.rs-2974671/v1

**Published:** 2023-06-06

**Authors:** Jennifer M. Frost, Jaehoon Lee, Ping-Hung Hsieh, Samuel J. H. Lin, Yunsook Min, Matthew Bauer, Anne M. Runkel, Hyung-Taeg Cho, Tzung-Fu Hsieh, Robert L. Fischer, Yeonhee Choi

**Affiliations:** Department of Plant and Microbial Biology, University of California, Berkeley; Department of Biological Sciences, Seoul National University; Department of Plant and Microbial Biology, University of California, Berkeley; Department of Plant and Microbial Biology, University of California, Berkeley; Department of Biological Sciences, Seoul National University; Department of Plant and Microbial Biology, University of California, Berkeley; Department of Plant and Microbial Biology, University of California, Berkeley; Department of Biological Sciences, Seoul National University; Department of Plant and Microbial Biology, North Carolina State University; Department of Plant and Microbial Biology, University of California, Berkeley; Department of Biological Sciences, Seoul National University

**Keywords:** Epigenetics, DNA demethylation, H2A.X, DEMETER, DNA damage response, DNA glycosylase

## Abstract

**Background:**

H2A.X is an H2A variant histone in eukaryotes, unique for its ability to respond to DNA damage, initiating the DNA repair pathway. H2A.X replacement within the histone octamer is mediated by the FAcilitates Chromatin Transactions (FACT) complex, a key chromatin remodeler. FACT is required for DEMETER (DME)-mediated DNA demethylation at certain loci in *Arabidopsis thaliana* female gametophytes during reproduction. Here, we sought to investigate whether H2A.X is involved in DME- and FACT-mediated DNA demethylation during reproduction.

**Results:**

H2A.X is encoded by two genes in Arabidopsis genome, *HTA3* and *HTA5*. We generated *h2a.x* double mutants, which displayed a normal growth profile, whereby flowering time, seed development, and root tip organization, S-phase progression and proliferation were all normal. However, *h2a.x* mutants were more sensitive to genotoxic stress, consistent with previous reports. H2A.X fused to Green Fluorescent Protein (GFP) under the *H2A.X* promoter was highly expressed especially in newly developing Arabidopsis tissues, including in male and female gametophytes, where DME is also expressed. We examined DNA methylation in *h2a.x* developing seeds and seedlings using whole genome bisulfite sequencing, and found that CG DNA methylation is decreased genome-wide in *h2a.x* mutant seeds. Hypomethylation was most striking in transposon bodies, and occurred on both parental alleles in the developing endosperm, but not the embryo or seedling. *h2a.x*-mediated hypomethylated sites overlapped DME targets, but also included other loci, predominately located in heterochromatic transposons and intergenic DNA.

**Conclusions:**

Our genome-wide methylation analyses suggest that H2A.X could function in preventing access of the DME demethylase to non-canonical sites. Alternatively, H2A.X may be involved in recruiting methyltransferases to those sites. Overall, our data suggest that H2A.X is required to maintain DNA methylation homeostasis in the unique chromatin environment of the Arabidopsis endosperm.

## Background

DNA methylation regulates gene expression and silences transposable elements (TEs) in plants and vertebrates [1], and epigenetic reprogramming by DNA demethylation is vital for reproduction in mammals and flowering plants [2–5]. In Arabidopsis thaliana, DNA demethylation during reproduction is catalyzed by the DNA glycosylase DEMETER (DME) [6]. DME is a dual function glycosylase/AP lyase, which actively removes DNA methylation via the Base Excision Repair (BER) pathway [7].

DME-mediated DNA demethylation occurs genome-wide at discrete loci that fall into two groups. The first consists of relatively euchromatic, AT-rich, small TEs that are nucleosome-poor, and generally interspersed with genes in chromosome arms [8]. The second group of loci require the Facilitates Chromatin Transactions (FACT) complex for DME access, and are longer, heterochromatic TEs prevalent in pericentromeric, gene poor regions, enriched with heterochromatic histone marks and H1 linker proteins [9]. DME and DME-FACT mediated DNA demethylation occurs specifically in male and female gamete companion cells, the vegetative and central cells, respectively [8, 10], and is vital for Arabidopsis reproduction, whereby loss of maternal DME or FACT results in development abnormalities, loss of genomic imprinting and seed abortion [6–8, 11, 12].

FACT is required for several other vital cellular functions, including transcription initiation and elongation, nucleosome assembly and disassembly, and for histone variant exchange, specifically of H2A.X [13–16]. In Arabidopsis, H2A.X is essential for the response to DNA damage, whereby the phosphorylation of its SQEF motif by Ataxia Telangiectasia Mutated (ATM) and ATR kinases, serves as a signal for recruitment of DNA repair and checkpoint proteins [16–18]. It is not known how FACT is recruited to DME target sites, and the apurinic/apyrimidinic (AP) sites created during base-excision repair (BER) can lead to the formation of double strand breaks [19]. We therefore sought to explore whether recruitment of H2A.X to sites of DME activity during BER may provide a functional link between H2A.X, FACT and DME during Arabidopsis reproduction. In order to investigate this, we analyzed the expression and activity of H2A.X during Arabidopsis reproduction, finding that H2A.X is expressed throughout the plant, particularly in developing tissues and the male and female gametophytes. The loss of H2A.X does not impair DME-mediated DNA demethylation, however, but leads to CG hypomethylation at intergenic regions and transposable elements, specifically in the endosperm.

## Results

### Arabidopsis seedlings lacking H2A.X have reduced DNA damage tolerance

**Figure 1:**
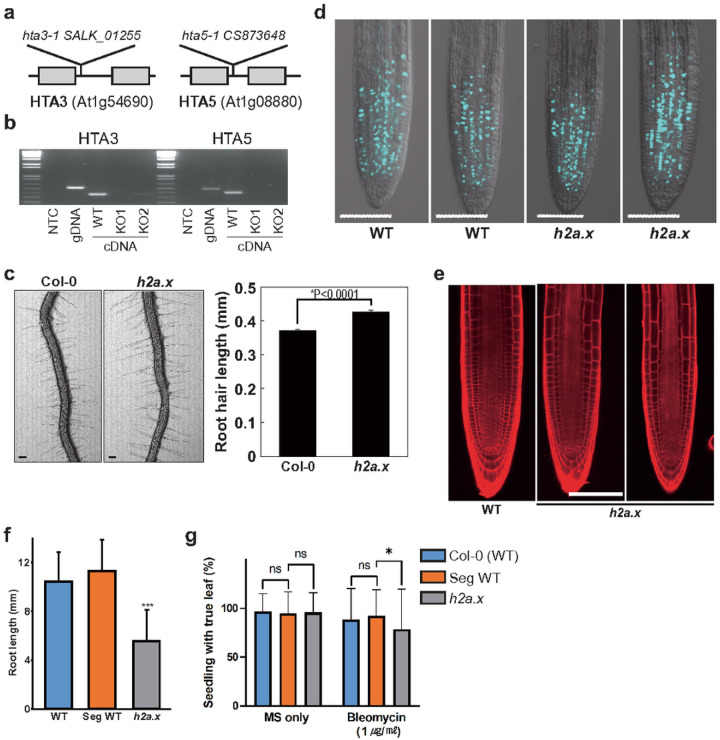
h2a.x mutant phenotype analysis. **a** HTA.3 and HTA.5 genomic loci, showing gene structure and location of T-DNA insertions. **b** The cropped image of qPCR analysis of each mutant, showing cDNA-specific PCR amplification and loss of gene product in mutant seedling tissue. **c** Root hair phenotypes of wild-type (Col-0) and *h2a.x* mutant primary roots and length measurements in mm. Data represent mean ± SEM (n = 1,419 root hairs for Col-0 and 1,159 root hairs for h2a.x from 35 ~ 40 roots. The asterisk (*) indicates a significant difference (Student’s t test). Scale bar, 100 μm. **d**EdU staining of WT and *h2a.x* double mutant roots at 3 DAG. Scale bar, 100 μm. **e** Propidium iodide (PI) staining of WT and *h2a.x* double mutant roots. Scale bar, 100 μm. **f** Aberrant root growth of *h2a.x*mutant seedlings when grown in bleomycin MS. Root length measurements are in mm and the result of three replicate experiments, each with 15 seedlings. **g**The formation of true leaves was slightly reduced in *h2a.x* mutant seedlings when grown in bleomycin MS. Measurements are the result of duplicated experiments. Leaves counted are; 415 in MS only and 203 in bleomycin MS for Col-0, 395 in MS only and 209 in bleomycin MS for H2A.X internal segregated WT control, 406 in MS only and 202 in bleomycin MS for *h2a.x* mutant. The box height and whisker length indicate the mean and standard deviation of each sample, respectively. The significance of differences between samples was measured by the Kolmogorov-Smirnov test. ns, not significant; * *p*=0.0440

*H2A.X* is encoded by two genes in Arabidopsis, *HTA3* (AT1G54690) and *HTA5* (AT1G08880). To investigate the effect of *H2A.X* mutations, we generated double mutants lacking both *HTA3* and *HTA5* (Fig. 1a), verified the loss of transcripts using RT-PCR (Fig. 1b and Additional file 1: Fig. S1a) and analyzed the sporophytic and gametophytic phenotypes of *h2a.x* plants. *h2a.x* mutant allele segregation, plant morphology, growth rate and flowering time were all normal, except for a significant increase in root hair length (Fig. 1c, p < 0.0001). *h2a.x* root hairs were ~ 15% longer than WT three days after germination (DAG). We then tested whether cell proliferation was normal in *h2a.x*, using the 5-ethynyl-2′-deoxyuridine (EdU), a thymine analog, and click chemistry to measure incorporation in newly synthesized DNA [20]. We did not observe a difference in EdU-stained cells between wild-type and h2a.x roots (Fig. 1d), indicating that S-phase progression and cell proliferation are normal in h2a.x mutants. We also measured whether there was increased DNA damage in mutant roots using propidium iodide (PI) staining but did not observe any differences between WT and *h2a.x* (Fig. 1e). These observations are consistent with mutant phenotypes observed in other DNA damage pathway genes, such as ATM or ATR kinases, which only exhibit a phenotype under growth conditions that promote DNA damage [21].

We therefore grew *h2a.x* and segregating WT seeds on MS plates containing Bleomycin sulphate, which induces double strand breaks (DSB) in DNA. MS Bleomycin concentrations of 0.5 ng/ml were used to test primary root formation and 1 ug/ml to test true leaf formation, as root development was more sensitive to the drug. *h2a.x* mutant seedlings had a significant reduction in root length compared to WT (Fig. 1f and Additional file 1: Fig. S1b). True leaf formation rate was slightly reduced in *h2a.x* mutants (Fig. 1g). These data are consistent with previous findings, also showing aberrant true leaf and root growth in *h2a.x* double mutant seedlings grown under genotoxic stress [22]. Thus, a lack of *H2A.X* resulted in increased sensitivity of developing tissues to DNA damaging agents, showing that *H2A.X* is required for the response to DNA damage in Arabidopsis.

### H2A.X is widely expressed across Arabidopsis tissues, including in gamete companion cells

**Figure 2:**
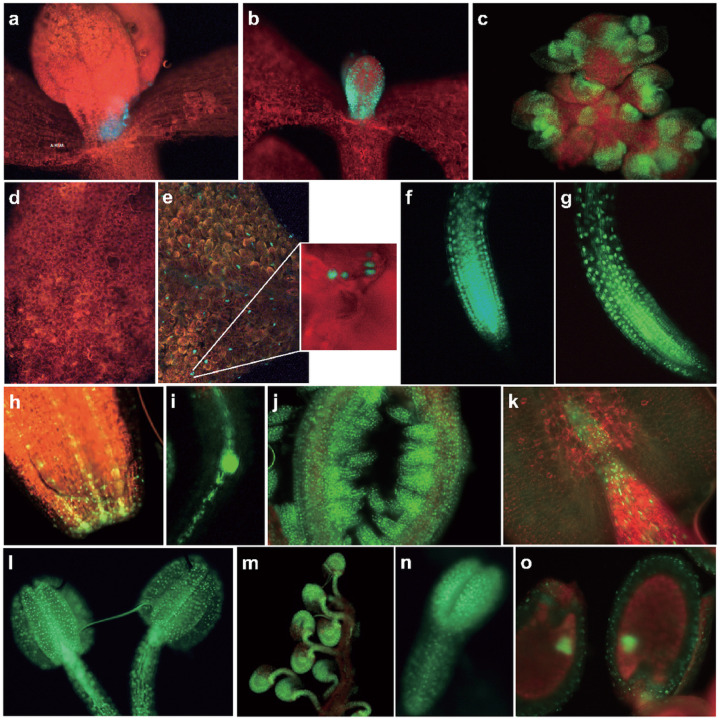
HTA5 expression is more dominant in dividing cells of the sporophyte. Both HTA5 and HTA3 fused to GFP showed expressions in the first true leaves (**a** and **b**), the floral meristem (**c**), the adaxial leaf surface (**d** and **e**), root tips (**f** and **g**), petal tips (**h**), and secondary root meristems (i). In reproductive structures supporting gametophyte development such as the ovule primordia (**j**) and anthers (**k and l**), and ovules (**m**) both isoforms were expressed. In the next generation seeds, both isoforms were present in the developing embryos (**n** and **o**).

**Figure 3:**
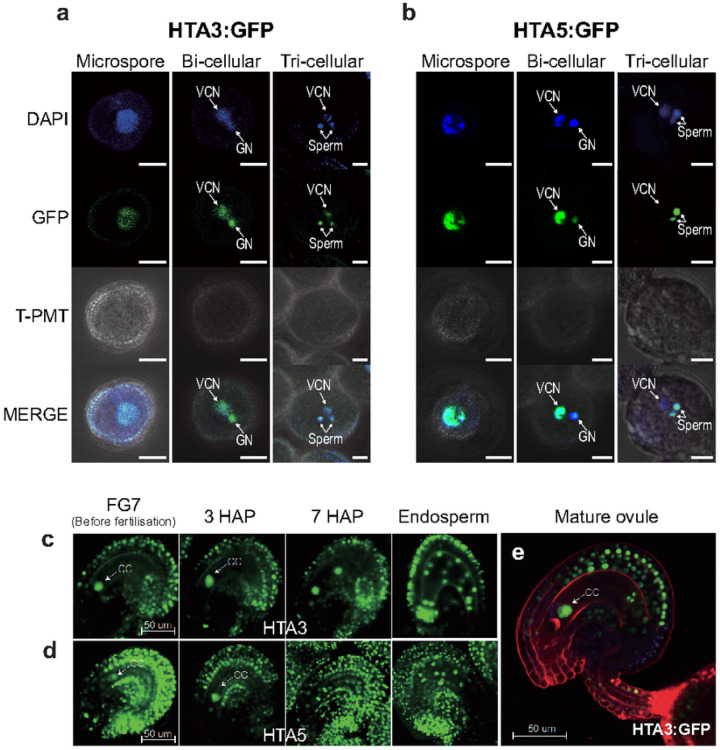
HTA3 expression is more dominant in gametophytic development. In the male gametophyte, both HTA3 and HTA5 are present in the microspore prior to mitosis. After Pollen Mitosis I (PMI) HTA3 was expressed in the generative and vegetative nucleus of bicellular pollen, and following Pollen Mitosis II (PMII), in both sperm cells and the vegetative nucleus of mature, tricellular pollen (**a**). HTA5 expression was also present in both the generative and vegetative nucleus following PMI, but was lost in the vegetative nucleus following PMII, in tricellular pollen (**b**). In the female gametophyte, egg cell expression was visible for both HTA3 and HTA5, but was weak. Conversely, HTA3 expression was very striking in the central cell, where it persisted following fertilization in the first cell divisions of the developing endosperm (**c** and **e**). HTA5 expression was also observed in the central cell, but expression in the surrounding ovule tissue was more prominent for this isoform (**d**).

To investigate the role of *H2A.X* in Arabidopsis development, we analyzed its expression pattern in sporophytic and reproductive tissues. We generated translational fusion constructs between the *Green Fluorescent Protein (GFP)* gene and either the *HTA3* or *HTA5* genes, including their promoter sequences, and introduced them into WT Col-0 Arabidopsis plants using Agrobacterium mediated transfer, deriving three and four independent lines for each allele, respectively. GFP fluorescence was observed using confocal microscopy. Both HTA3 and HTA5 proteins were expressed in dividing cells of the sporophyte: First true leaves (Fig. 2a and b), the floral meristem (Fig. 2c), the adaxial leaf surface (Fig. 2d and e), root tips (Fig. 2f and g), petal tips (Fig. 2h) and root meristem (Fig. 2i), though HTA5 was more strongly expressed. In reproductive structures supporting gametophyte development, such as the ovule primordia (Fig. 2j), anthers (Fig. 2k and l), and developing ovules (Fig. 2m), HTA5 was predominant. In the next generation seed, both isoforms were present in the developing embryo (linear cot), but not in endosperm at this stage (heart) (Fig. 2n and o). Conversely, in gametophytic development, both isoforms were again expressed but HTA3 was the dominant isoform (Fig. 3). In the male gametophyte, both HTA3 and HTA5 were present in the microspore prior to mitosis. After Pollen Mitosis 1 (PMI) HTA3 was expressed in the generative and vegetative nucleus of bicellular pollen, and following Pollen Mitosis II (PMII), in both sperm cells and the vegetative nucleus of mature, tricellular pollen (Fig. 3a). HTA5 expression was also present in both the generative and vegetative nucleus following PMI, but was lost in the vegetative nucleus following PMII, in tricellular pollen (Fig. 3b). In the female gametophyte, egg cell expression was visible for both HTA3 and 5, but was weak, conversely, HTA3 expression was very striking in the central cell, where it persisted following fertilization in the first cell divisions of the developing endosperm (Fig. 3c and e). HTA5 expression was also observed in the central cell, but expression in the surrounding ovule tissue was more striking for this isoform (Fig. 3d).

### DME does not regulate H2A.X expression in the Arabidopsis gametophyte

**Figure 4:**
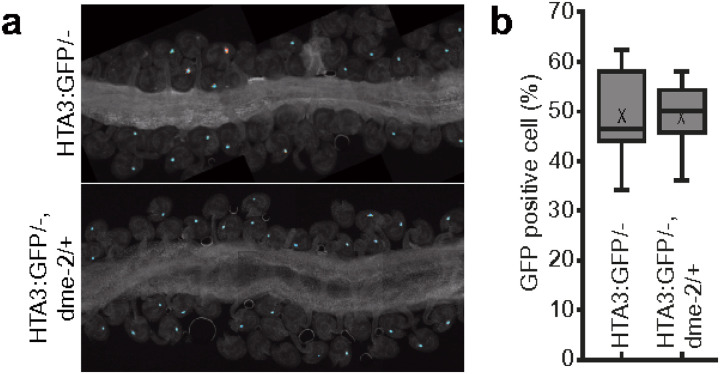
HTA3:GFP expression in wild-type and dme-2 mutant central cells. Confocal image of WT (a) and *dme-2* mutant (b) developing ovules expressing an HTA.3:GFP transgene. Expression is confined to the central cell. **c** Box plot showing the distribution of GFP positive central cells between WT and *dme-2* mutant ovules. There was no significant change in GFP positive central cells in *DME/dme-2*. + mark in boxplot is mean of data. The line in a box is median (n = 554 mature ovules for HTA3:GFP/- plants and 412 mature ovules for HTA3:GFP/- in *DME/dme-2*heterozygous plants. The values are not significantly different by Student’s t test.

Since *H2A.X* expression was prominent in the central and vegetative cells, specifically where DME-mediated demethylation and related BER activity takes place [10, 23], we reasoned that *H2A.X* expression may be regulated by promoter DNA methylation, whereby DME might demethylate *HTA3* and *HTA5* promoter sequences in the gametophyte, increasing expression of these transcripts. To test this hypothesis, we utilized wild-type plants hemizygous for the *HTA3:GFP* transgene, for which strong HTA3 expression could be observed in the central cell in ~ 50% of the developing ovules (Fig. 4). We crossed these plants with DME/*dme*-2 heterozygotes to derive DME/*dme*-2 plants that were also hemizygous for the *HTA3:GFP* transgene. A maternally inherited *dme*-2 mutation generates embryo abortion and seed lethality, so analysis of seed development is generally only possible in DME/*dme-2* heterozygotes. We then analyzed the incidence of HTA3:GFP expression in DME/*dme-2* mutants and their segregating wild-type siblings in the F2 population. In both DME/DME HTA3:GFP/- (Fig. 4a) and DME/*dme*-2 HTA3:GFP/-F2 (Fig. 4b) siblings we observed that ~ 50% of the female gametophytes within ovules produced a strong GFP signal, indicating that the loss of DME did not alter the expression of *H2A.X* in the Arabidopsis female gametophyte. Consistent with this, when we compared promoter DNA methylation for the H2A.X variants in Arabidopsis wild-type and *dme-2* mutant central cells and endosperm [11, 24], we found that *H2A.X* promoter methylation was low in both tissues, and unchanged in the *dme*-2 mutant (Additional file 1: Fig. S2a and S2b). Other H2A variant gene loci were also unmethylated in both wild-type and *dme-2* mutant central cell and endosperm, except for H2A.Z.4, which exhibited promoter methylation in central cell and endosperm, that increased in *dme-2* mutants, a hallmark of a DME-target promoter (Additional file 1: Fig. S2c).

### h2a.x mutant endosperm is hypomethylated genome-wide

**Figure 5:**
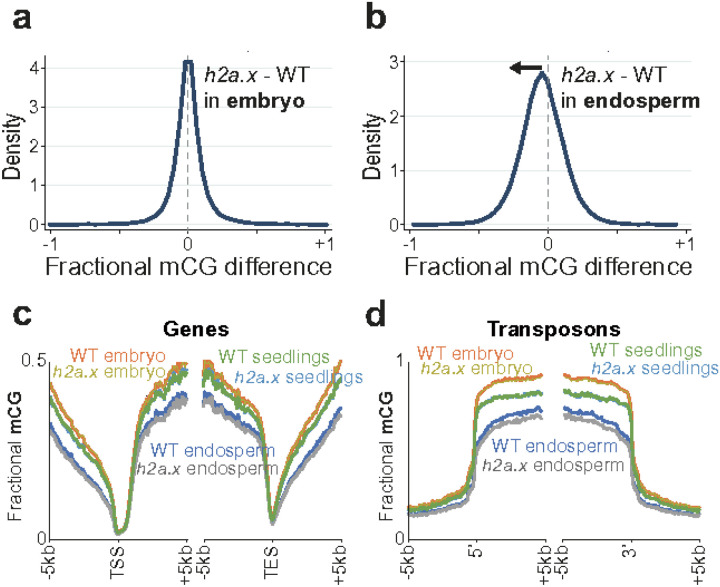
Genome-wide methylation analysis of selfed double *h2a.x* mutant developing embryo, endosperm and seedling. **a** Fractional methylation difference between *h2a.x* double mutant and WT CG methylation from embryo (linear-bending cotyledon) is plotted, data in 50 bp windows with >10x sequence coverage. Data are from *h2a.x* Col selfed plants and segregating wild type siblings. **b** As for **a**, but with endosperm. **c** Ends analysis of *h2a.x* mutant genomic methylation in genes, with genes aligned according to their 5′ and 3′ ends. **d** Ends analysis of *h2a.x*mutant genomic methylation in transposons, with transposons aligned according to their 5′ and 3′ ends.

To investigate whether changes in DNA methylation were present in *h2a.x*, we carried out genome-wide bisulfite sequencing (BS-seq) of manually dissected endosperm and embryo from homozygous *h2a.x* mutant and wild-type F1 seeds and their resulting seedlings, following self-pollination of homozygous *h2a.x* mutants and wild-type sibling plants. We observed that embryo DNA methylation in the *h2a.x* mutant was unchanged from wild-type, with the peak of fractional methylation difference at zero (Fig. 5a). However, DNA methylation of *h2a.x* mutant endosperm was reduced compared to wild-type in the CG context, with the fractional methylation difference peak shifted to the left (Fig. 5b).

To ascertain which endosperm loci were hypomethylated, we aligned our methylome data to the 5′ transcriptional start sites (TSS) and 3′ transcriptional end sites (TES) of genes and transposons, also including the *h2a.x* seedling methylome. Hypomethylation was present only in endosperm, and although also present in gene bodies and intergenic regions, was most striking in transposon bodies, (Fig. 5c and d). CHG and CHH methylation was also reduced in endosperm transposon bodies (Additional file 1: Fig. S3a-S3d). In *h2a.x* embryos, CHH methylation in TEs was decreased (Additional file 1: Fig. S3d). However, embryo CHH methylation increases steadily with time during embryo development [25] so it is likely the differences observed are technical, whereby mutant seeds were dissected slightly earlier in their development than wild-type. Consistent with this, CHH methylation in *h2a.x* seedlings, which derive directly from the embryo, was not different from WT (Additional file 1: Fig. S3d).

## H2A.X hypomethylation overlaps DME target loci

**Figure 6:**
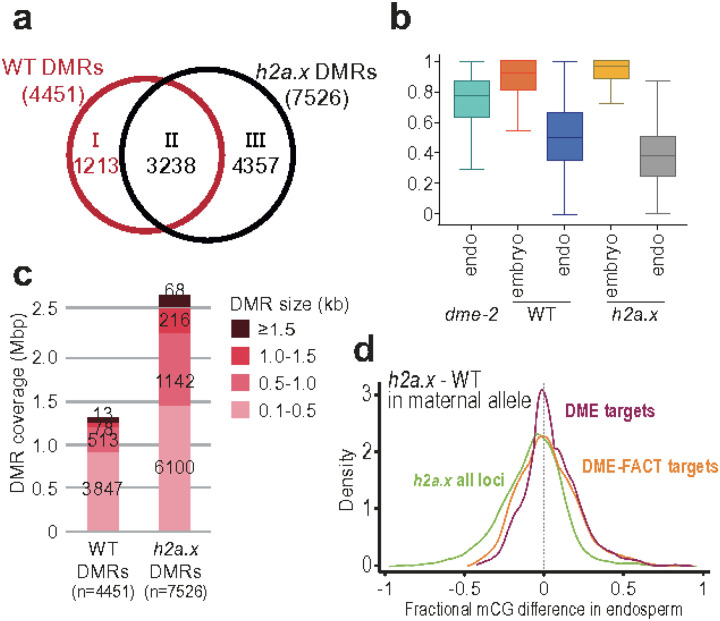
Endosperm-Embryo hypomethylated DMR analysis in WT and h2a.x mutants. Analysis of *h2a.x*methylomes, comparing DMRs between endosperm and embryo. **a** Venn diagram illustrating that WT embryo and endosperm harbour 4451 DMRs, the majority of which (3238) are shared with *h2a.x* embryo and endosperm. *h2a.x*embryo and endosperm have an additional 4357 DMRs. **b** Box plots showing the relative methylation level of DMRs in embryo and endosperm, in wild type, *h2a.x*and *dme-2* mutants. **c** Characterization of *h2a.x*-specific embryo-endosperm DMRs; wild-type and *h2a.x* Endosperm-Embryo DMRs grouped by size, with the cumulative total length they covered shown, whereby they are represented across all DMR sizes, and represent an overall increase in size distribution**. d** Fractional methylation difference between *h2a.x* mutant and WT CG methylation from the maternal endosperm allele is plotted, data in 50 bp windows with >10x sequence coverage, for all loci (green), DME target loci (red) and DME-FACT target loci (orange)

Inheritance of a maternal loss-of-function *dme* allele or a maternal loss-of-function *ssrp1* allele, which encodes one of the proteins in the FACT complex, result in striking phenotypes of seed abortion and developmental delay. Seed viability in homozygous *h2a.x* mutants, as well as in crosses from maternal *h2a.x* with wild-type Col-0 pollen, was normal, suggesting that DME- and DME-FACT- mediated DNA demethylation occurred normally in *h2a.x* mutant seeds, at least at PRC2 genes critical for seed viability. In wild type female gametophytes, the central cell genome undergoes genome-wide epigenetic remodeling. DME/DME-FACT-mediated DNA demethylation leads to a deeply hypomethylated maternal endosperm genome compared to embryo [8, 9, 11, 24]. To assess whether *h2a.x* mutant hypomethylated regions overlapped sites of canonical endosperm hypomethylation, we compared differential methylated regions between endosperm and embryo (EN-EM DMRs) in WT and *h2a.x* mutant seeds. There were 4,451 hypo-EN-EM DMRs between WT endosperm vs embryo, covering about 1.3 M bps. In contrast, 7,526 hypomethylated EN-EM DMRs were identified between *h2a.x* endosperm and embryo, covering 2.7 M bps in length, more than double the area of the wild-type hypomethylated EN-EM DMRs (Fig. 6a).

Since many wild-type EN-EM DMRs are a result of DME activity in the central cell, we measured the overlap between *h2a.x* EN-EM DMRs and DME targets, finding that of the 7526 *h2a.x* EN-EM DMRs, 4692 (62%) overlapped with canonical DME target loci [8]. We therefore delineated the hypomethylated *h2a.x* EN-EM DMRs according to whether they overlapped WT embryo-endosperm DMRs (including DME targets) (n = 3238), and those that were novel *h2a.x* specific DMRs (n = 4357; Fig. 6a and b). There was also a group of WT DMRs which were only differentially methylated between WT embryo and endosperm (n = 1213). We delineated DMRs by size (0.1 kb->1.5 kb) and found that *h2a.x* EN-EM DMRs were represented across all size classes, with similar proportions of size classes to wild-type (Fig. 6c).

Since *h2a.x* hypomethylation overlapped DME targets, we next assessed whether the *h2a.x* mutation affected DME activity in the central cell. Maternal *h2a.x* mutant plants in the Columbia ecotype were pollinated with wild-type L*er* pollen, and BS-seq with embryo and endosperm from manually-dissected mutant and segregating wild-type seeds carried out, sorting resulting reads according to their parental ecotype. In this way, the maternal endosperm genome can be used as a proxy for the central cell genome. Here, the kernel density methylation plot peak is close to zero, but slightly negative, indicating that the maternal endosperm genome is hypomethylated (Fig. 6d, green trace and Fig. 5b). We then specifically looked at maternal DME and shared DME-FACT loci, but these peaks also lay close to zero, with no striking skewing observed (Fig. 6d, red and orange traces, respectively). This indicates that whilst *h2a.x* hypomethylated loci do overlap some DME and DME-FACT targets, the *h2a.x* mutation does not affect DME/FACT targeting or activity at their canonical sites.

### h2a.x mutant endosperm hypomethylation occurs post-fertilisation

**Figure 7:**
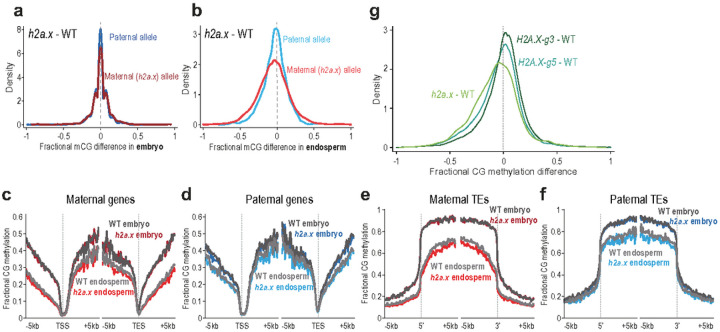
Methylome analysis of seeds h2a.x maternal and WT paternal plant crosses. Genome-wide methylation analysis of *h2a.x* mutant developing embryo and endosperm, comparing maternal and paternal alleles in wild-type (WT) and *h2a.x* mutant crosses whereby the maternal allele is either WT Columbia or *h2a.x* homozygous mutant Columbia, and the paternal allele is always wild-type L*er*; ‘*h2a.x* paternal’ denotes a wild-type paternal allele now resident in a heterozygous *h2a.x* mutant seed. **a** Fractional methylation difference between *h2a.x* double mutant gametophyte crossed with L*er*WT pollen in CG methylation from embryo (linear-bending cotyledon) is plotted, data in 50 bp windows with >10x sequence coverage. **b** As for **a**, but with endosperm. A slight shift towards the left can be seen for the maternal endosperm allele (inherited from *h2a.x* mutant central cell). **c**Ends analysis of maternal (*h2a.x* mutant) genomic methylation in genes, with genes aligned according to their 5′ and 3′ ends. **d** Ends analysis of paternal genomic methylation in genes, with genes aligned according to their 5′ and 3′ ends. **e** Ends analysis of maternal (*h2a.x* mutant) genomic methylation in transposons, with transposons aligned according to their 5′ and 3′ ends**. f** Ends analysis of paternal genomic methylation in transposons, with transposons aligned according to their 5′ and 3′ ends. **g** Fractional CG DNA methylation difference between mutant and WT maternal endosperm in developing seeds from both *hta3/hta3 hta5/+* (*H2A.X-g3*) and *hta5/hta5 hta3/+* mutants (*H2A.X-g5*) were crossed to L*er*, so that the sporophyte had one remaining copy of one of the isoforms, but both H2A.X isoforms are lost in ½ of the gametophytes. *H2A.X-g5*and *H2A.X-g3* are plotted alongside *h2a.x*. For both *H2A.X-g3*and *H2A.X-g5*, the curve peaks are close to zero, whereas the full *h2a.x*mutant is skewed to the left, indicating redundancy between HTA5 and HTA3 isoforms in the context of mutant endosperm DNA hypomethylation.

Since the maternal *h2a.x* endosperm allele was hypomethylated, yet H2A.X is not expressed in wild type endosperm, (Fig. 2p) we next sought to investigate whether *h2a.x*-mediated methylation loss originated in the maternal gametophyte. Consistent with this idea, both *H2A.X* isoforms are strongly expressed in the wild-type central cell (Fig. 3c and d). Using data from the F1 crosses outlined above, where maternal *h2a.x* mutant plants in the Col-0 ecotype were pollinated with wild-type L*er* pollen, we next plotted both maternal and paternal CG methylation. In *h2a.x* embryos, both maternal and paternal allele CG methylation is identical to WT (peak aligns on zero, Fig. 7a), consistent with our observations in self-pollinated *h2a.x* mutants (Fig. 5a). However, in endosperm, a slight shift is visible towards the left, indicating mutant hypomethylation is present on both maternal and paternal endosperm alleles (Fig. 7b). This indicates that whilst maternal allele hypomethylation may be inherited from the central cell, hypomethylation of the wild type paternal allele is also present. Paternal allele hypomethylation must manifest post-fertilization, i.e. due to a reduction in CG methylation efficiency or maintenance. To ascertain which parental loci were hypomethylated, we again aligned our methylomes to the TSS/5′ and TES/3′ ends of genes and transposons (Fig. 7c–f and Additional file 1: Fig. S4a-S4d). As in the previous analysis, CG methylation in embryo is not different from wild-type in heterozygous *h2a.x* mutant gene and transposon bodies, but both maternal and paternal endosperm alleles are hypomethylated in genes, intergenic regions and TEs, with hypomethylation in TE bodies being most visible. CHG methylation is the same in wild-type and *H2A.X/h2a.x* mutant embryo and endosperm (Additional file 1: Fig. S4e-S4h) whereas CHH methylation is decreased on both parental alleles, in both *H2A.X/h2a.x* embryo and endosperm (Additional file 1: Fig. S4i-S4l).

*H2A.X* is encoded by two almost identical isoforms, HTA3 and HTA5. To determine whether one isoform may have an effect independent of the other, we dissected developing seeds from both *hta3/hta3 hta5/+* (*H2A.X-g3*) and *hta3/+ hta5/hta5* mutants (*H2A.X-g5*), crossed to L*er*, so that the sporophyte had one remaining copy of one of the isoforms, but both *H2A.X* isoforms are lost in ½ of the gametophytes. Following BS-seq, we determined that both isoforms act redundantly, whereby endosperm methylation was not substantially affected in either *hta3/hta3 hta5/+* or *hta5/hta5 hta3/+* seeds (Fig. 7g, H2A.X-g3 and H2A.X-g5, kdensity peaks on zero) compared to hypomethylated *h2a.x* double mutant endosperm (Fig. 7g, h2a.*x* peak shifted to the left).

### h2a.x hypomethylation is widespread in intergenic DNA

**Figure 8:**
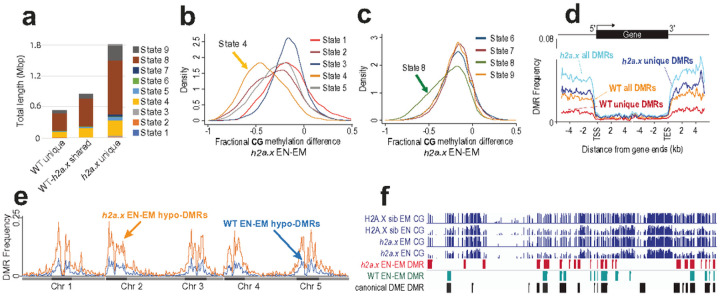
Chromatin states of *h2a.x* DMRs. **a** Comparison of the chromatin states comprising each group of DMRs from Fig. 6a. Chromatin state distribution, and the total length they covered, within WT unique, WT-*h2a.x*shared, and *h2a.x* unique Endosperm-Embryo DMRs. States 1 to 7 correspond to euchromatin, and states 8 and 9 correspond to AT- and GC-rich heterochromatin, respectively. The chromatin states most increased (as a fraction of their total) in *h2a.x* embryo-endosperm DMRs are 4, 8 and 9. (**b** and **c**) Kernel density plots showing the fractional methylation difference for *h2a.x* mutant endosperm (EN) minus embryo (EM), plotted according to chromatin state, demonstrating that the largest shift to endosperm hypomethylation (i.e. to the left) lies in State 4 (yellow in **b**) and State 8 (green in **c**). **d** Ends analysis plot showing distribution frequency of DMRs with respect to coding genes. Genes were aligned at the 5′- or the 3′-end, and the proportion of genes with DMRs in each 100-bp interval is plotted. DMR distribution is shown with respect to all WT DMRs (orange trace), WT unique DMRs (red trace), all *h2a.x*DMRs (cyan trace), and *h2a.x* unique DMRs (blue trace). **e** Arabidopsis chromosome view of genome-wide methylation levels for *h2a.x* mutant DMRs between endosperm (EN) and embryo (EM), and WT DMRs between endosperm (EN) and embryo (EM), represented by the distribution frequency of DMRs along the 5 chromosomes. Dark blocks represent centromere and peri-centromeric regions of each chromosome. **f** IGV browser view of methylome data and DMR calls for *H2A.X* segregating wild-type endosperm and embryo (Green), *h2a.x* mutant endosperm and embryo (Red), as well as DMRs identified between *dme-2 /wt* endosperm (Black) [8].

To assess if *h2a.x* endosperm hypomethylated loci are associated with particular chromatin states, we used published histone marks and genomic characteristics that topologically group the Arabidopsis genome into nine distinct chromatin states [26] and used them to compare methylation differences between homozygous *h2a.x* vs wildtype endosperm. For the hypomethylated EN-EM DMRs specific to *h2a.x*, the majority reside in non-coding, intergenic sequences, including distal promoters (chromatin state 4, Fig. 8a) and AT-rich heterochromatic regions (chromatin states 8 and 9, Fig. 8a), consistent with what we observed in TE metaplots (Fig. 5c and d, Fig. 7e and f). In addition, when we used fractional methylation differences to analyse the chromatin states of hypomethylated loci unique to the *h2a.x* mutant, i.e. not including those present in between wild-type embryo and endosperm, chromatin states 4 and 8 exhibit the largest shift (Fig. 8b and c). These data indicate that the novel, *h2a.x*-specific EN-EM DMRs lie primarily in chromatin states 4, 8 and 9.

In order to gain resolution on DMR location, we aligned wild-type and *h2a.x* DMR coordinates according to 5′ and 3′ ends of genes, which revealed that *h2a.x* hypomethylation is enriched in intergenic regions, consistent with its enrichment in chromatin states 4 and 8 (Fig. 8d). To further characterize the location of *h2a.x* EN-EM DMRs, we plotted their co-ordinates across the Arabidopsis genome in 300 kb bins (see Methods and Materials; Fig. 8e). This analysis showed that *h2a.x* EN-EM DMRs in general mirror the distribution of wild-type EN-EM DMRs, which are enriched in pericentric heterochromatin (Fig. 8e). To determine whether the *h2a.x* endosperm hypomethylation represented novel sites of demethylation, or resulted from increased demethylation at already demethylated sites (e.g., resulting in longer DMRs), we took a locus-specific approach, using the IGV genome browser to view aligned methylation data and DMRs (Fig. 8f). The majority of *h2a.x*-specific hypomethylation represented stand-alone, novel DMRs (red outline). *h2a.x* DMRs overlapped DME-mediated wild-type endosperm/embryo DMRs (green outline), but did not make them longer.

## Discussion

*H2A.X* is one of the H2A variants in higher eukaryotes and differs from canonical H2A by its rapid phosphorylation to y-*H2A.X* in response to DNA double-strand breaks. Unmodified *H2A.X* is ubiquitously expressed and distributed throughout the genome as a component of nucleosome core structure, estimated to represent approximately 10% of H2A variants present in chromatin at any given time [27–30]. We show that H2A.X is widely expressed in Arabidopsis newly developing tissues and reproductive cells, including the companion cells of the male and female gametophytes the vegetative and central cells, respectively. Loss of H2A.X results in endosperm hypomethylation at intergenic regions and in heterochromatic TEs.

It was previously shown that the *HTA3* and *HTA5* gene promoters exhibited differences in activity, with *HTA5* observed to be less active in the floral bud [31]. Consistent with this, we show that whilst HTA5 is the predominant protein isoform expressed in the sporophyte, HTA3 predominates in the developing gametophytes, though both are highly expressed in pollen. One explanation for H2A3/5 high expression in the vegetative and central cells is that DME activity creates AP sites during BER, that may lead to the formation of double strand breaks, thereby requiring high levels of H2A.X [19]. Intriguingly, however, in heterochromatin, the mechanism of DNA repair is different; an H2A.W variant, H2A.W.7 is phosphorylated by ATM to initiate the response in constitutive heterochromatin to DNA damage [22].

We observed a significant increase in root hair length in *h2a.x* mutants compared to wild-type in the absence of any DNA damaging conditions. Intriguingly, reduction in H2A.Z incorporation into chromatin also results in an increase in root hair length, since the altered chromatin state mimics phosphate deficiency - activating a phosphate deficiency response gene locus [32, 33]. Similarly, *h2a.x* mutations may indirectly affect the expression of root hair-growth genes [34–36]. Alternatively, defective *H2A.X* expression may also cause nutrient-stress, resulting in the modulation of genes involved in root hair growth.

We identified hypomethylation on both male and female endosperm alleles in hybrid maternal *h2a.x* mutants crossed to paternal wild-type F1, meaning that hypomethylation manifests post-fertilization, at least on the paternal allele. On the maternal allele, both pre- and post-fertilization DNA methylation dysregulation may be present. Hypomethylation was also confined to endosperm, whereas *h2a.x* embryo was normally methylated. Endosperm is a triploid tissue, with distinct higher-order chromatin structure compared to other tissues, being less condensed, and subsequently featuring increased trans-chromatin interactions, increased expression of TEs, and encroachment of heterochromatin into euchromatic regions [37, 38]. Endosperm is also the site of parental competition for generational resources, in part reflected in the activities of DME and FACT in the central cell, which confer deep hypomethylation. As such, the chromatin environment of the central cell and endosperm may in turn impact DNA methylation homeostasis in *h2a.x* null tissues differently to embryo.

Our *h2a.x* EN-EM DMR analyses revealed the following characteristics of *h2a.x*-specific hypomethylated DMRs: enriched in chromatin states 4, 8 and 9 [26] and located in TEs, pericentric heterochromatin and intergenic regions. Chromatin states 4 and 8 are strikingly enriched in intergenic DNA (66.2 and 58.2%, respectively, [26]. Chromatin state 4 is also characterized by the presence of histone variants H3.3 and H2A.Z, and high levels of H3K27me3, but is not highly associated with active transcription, and likely to contain distal promoters and regulatory elements. Chromatin states 8 and 9 are highly enriched in TEs, and feature H3.1, H3K9me2, and H3K27me1 enrichment, and although state 8 is a transitional, more decondensed state, they both represent Arabidopsis heterochromatin [26].

The mechanism of DNA methylation loss in *h2a.x* endosperm remains unclear. The *h2a.x* hypomethylated DMRs represent regions that are not normally demethylated during seed development. Potentially, H2A.X could function in wild-type cells to exclude DME from these regions; preventing inappropriate remodeling of regulatory DNA and heterochromatic TEs. As such, when H2A.X is lost, DME may demethylate further sites in the central cell, contributing to endosperm hypomethylation, specifically of the maternal allele. However, in human cells, H2A.X phosphorylation destabilises chromatin structure, increasing its accessibility [16]. Nucleosome cores are crucial for nuclear DNA organization and function, and lost H2A.X is likely quickly replaced by other H2A variants, such as H2A.Z, H2A.W, or by canonical H2A. The replacement of H2A.X with variants that cannot be phosphorylated, may therefore reduce chromatin access, for example, to DNA methyltransferases, resulting in comparative hypomethylation.

In conclusion, we demonstrate that *H2A.X* is expressed widely in developing Arabidopsis tissues and gamete companion cells, and show that the DNA damage response is impaired in *h2a.x* mutant roots and seedlings. We show that *h2a.x* mutant endosperm exhibits DNA hypomethylation at intergenic regions and heterochromatic TEs, creating a large number of endosperm-embryo DMRs, not present in wild-type. We speculate that *h2a.x* mediated hypomethylation may be due to an increase in DME targeting to non-canonical sites, or a loss of chromatin accessibility to methyltransferases.

## Conclusions

In this study, we examined the effect of *h2a.x* double mutants on genome-wide methylation patterns between embryo and endosprm, as well as between maternal and paternal alleles, compared to wild type. Hypomethylation was observed in the *h2a.x* endosperm, but not in the embryo or seedling. This hypomethylation was present on both maternal and paternal alleles, suggesting that hypomethylation occurs after fertilization, at least on paternal allele of *h2a.x* mutant endopserm. While the expression patterns and methylation targets of H2A.X ovelap with those of DME, other loci including heterochromatic transposons and intergenic sequences that are non-DME or non-DME-FACT canonical sites, are also hypomethylated in *h2a.x* mutant endopserm. In summary, our findings suggest that H2A.X palys a role in preserving the balance of DNA methylation within its unique target sites, which represent distinctive chomomation states in Arabidopsis endopserm.

## Methods and Materials

### Plant materials and growth conditions

Wild type and *h2ax* mutant Arabidopsis seeds were bleached and sown onto Murashige and Skoog plates, followed by cold treatement in the dark at 4 degrees C for 3 days, and two weeks growth in a light chamber, before transplantation onto soil. Seedlings were grown in a greenhouse with a long-day photoperiod (16 h light, 8 h dark). Seed stocks of T-DNA insertion mutants (SALK_012255 in HTA3 and SAIL_382_B11/CS873648 in HTA5, Fig. 1a) in the Columbia-0 (Col-0) background were obtained from the ABRC stock center. Mutant alleles were, backcrossed five times to wild-type, and finally crossed to obtain double *hta3/hta3; hta5/hta5* null plants, designated *h2a.x*, as well as segregating wild-type siblings. Both T-DNA insertion alleles have been studied and validated in recent reports [22, 39].

### Edu cell proliferation assay

5-ethynyl-2′-deoxyuridine (EdU) staining using an Invitrogen Click-iT^™^ EdU Alexa Fluor^™^ 488 HCS Assay (C10350) was performed based on Kotokany et al. (2010) [20] to detect S phase cells. Seeds were grown in MS media vertically for 3 days. Seedlings were collected in MS solution containing 1μM Edu and incubated at 22°C for 30 minutes. Samples were fixed in 4%(w/v) formaldehyde solution in phosphate-buffered saline (PBS) with 0.1% Triton X-100 for 30min, and washed three times with PBS each for 5 minutes. The samples were incubated in Edu detection cocktail solution at room temperature for 30 minutes in the dark, and washed with the Click-iT^®^ rinse buffer and then three times with PBS. The photos were taken using confocal microscopy (LSM700, Zeiss).

### Propidium Iodide (PI) staining

Propidium Iodide (PI, P-4170, sigma) staining was used to detect cell death and show anatomy of the roots. The samples were stained with working PI solution (5ml PI solution in 1ml of distilled water) at room temperature for 30s and washed with distilled water on slide glass.

### Observation of root hair phenotypes

A stereomicroscope (M205 FA, Leica) was used for the observation of root hair phenotypes. Root hair length was measured as previously described by [40] with slight modifications as in the [41].

#### H2A.X expression localization

HTA3 and HTA5 GFP fuson proteins were cloned alongside a hygromycin resistance casette using a Gibson assay (Invitrogen) and F1 seeds screened on MS containing hygromycin. F1 plants were screened manually using a fluorescence microscope and seeds collected from plants expressing GFP. F2 seeds were grown on hygromycin and selected if we identified segregation of the resistance allele, indicating the presence of a single copy transgene. F3 plants were then used for confocal microscopy.

### DNA damage assay

Segregating WT and *h2a.x* homozygous mutant Arabidopsis seeds were planted on MS containing 0.5ug/ml bleomycin sulphate and grown vertically for 14 days under long day conditions, before measuring root length. MS without bleomycin was used as a control. Values are from three independent experiments each including 15 seedlings for each genotype. True leaf assay was performed as previously described with 10-day-old seedlings [42].

### Isolation of Arabidopsis endosperm and embryos

Segregating WT and *h2a.x* mutant Arabidopsis flower buds were either allowed to self, or emasculated at flower stage 12–13 using fine forceps and pollinated with L*er* pollen 48 hours later. Eight to ten days after pollination (DAP) developing F1 seeds (linear to bending cotyledon stage) were immersed in dissection solution (filter-sterilized 0.3 M sorbitol and 5 mM pH 5.7 MES) on sticky tape and dissected by hand under a stereo-microscope using fine forceps (Fine Science Tools, Inox Dumont #5) and insect mounting pins. The seed coat was discarded, and debris removed by washing collected embryos or endosperm five to six times with dissection solution under the microscope.

### Bisulfite sequencing library construction

As described previously, genomic DNA was isolated from endosperm and embryo [11]. Bisulfite sequencing libraries for Illumina sequencing were constructed as in [8] with minor modifications. In brief, about 50 ng of genomic DNA was fragmented by sonication, end repaired and ligated to custom-synthesized methylated adapters (Eurofins MWG Operon) according to the manufacturer’s instructions for gDNA library construction (Illumina). Adaptor-ligated libraries were subjected to two successive treatments of sodium bisulfite conversion using the EpiTect Bisulfite kit (Qiagen) as outlined in the manufacturer’s instructions. The bisulfite-converted library was split between two 50 ul reactions and PCR amplified using the following conditions: 2.5 U of ExTaq DNA polymerase (Takara Bio), 5 μl of 10X ExTaq reaction buffer, 25 μM dNTPs, 1 μl Primer 1.1 and 1 μl multiplexed indexing primer. PCR reactions were carried out as follows: 95°C for 3 minutes, then 14–16 cycles of 95 °C 30 s, 65 °C 30 s and 72 °C 60 s. Enriched libraries were purified twice with AMPure beads (Beckman Coulter) prior to quantification with the Qubit fluorometer (Thermo Scientific) and quality assessment using the DNA Bioanalyzer high sensitivity DNA assay (Agilent). Sequencing on either the Illumina HiSeq 2000/2500 or HiSeq 4000 platforms was performed at the Vincent J. Coates Genomic Sequencing Laboratory at UC Berkeley.

### Bisulfite data analysis

Sequenced reads were sorted and mapped to Col-0 and L*er* genomes in cases of seeds derived from Col × L*er* crosses, or not sorted and mapped to Col-0 for selfed samples. Gene and TE ends analysis and kernel density plots were generated as previously described [8], using only windows with at least 10 informative sequenced cytosines, and fractional methylation of at least 0.5 (CG), 0.4 (CHG) or 0.08 (CHH) in at least one of the samples being compared. In addition, the bisulfite conversion rate was calculated using chloroplast DNA methylation ratio (Additional file 2: Table. S1).

## Data Availability

The datasets generated and analyzed during the current study are in the NCBI GEO under accession number GSE233920 (reviewer link; https://www.ncbi.nlm.nih.gov/geo/query/acc.cgi?acc=GSE233920).

## References

[1] Law JA, Jacobsen SE: Establishing, maintaining and modifying DNA methylation patterns in plants and animals. Nat Rev Genet 2010, 11(3):204–220. 20142834 10.1038/nrg2719 PMC3034103

[2] Monk M, Boubelik M, Lehnert S: Temporal and regional changes in DNA methylation in the embryonic, extraembryonic and germ cell lineages during mouse embryo development. Development 1987, 99(3):371–382. 3653008 10.1242/dev.99.3.371

[3] Feng S, Jacobsen SE, Reik W: Epigenetic reprogramming in plant and animal development. Science 2010, 330(6004):622–627. 21030646 10.1126/science.1190614 PMC2989926

[4] Parrilla-Doblas JT, Roldan-Arjona T, Ariza RR, Cordoba-Canero D: Active DNA Demethylation in Plants. Int J Mol Sci 2019, 20(19). 10.3390/ijms20194683 PMC6801703 31546611

[5] Wu X, Zhang Y: TET-mediated active DNA demethylation: mechanism, function and beyond. Nat Rev Genet 2017, 18(9):517–534. 28555658 10.1038/nrg.2017.33

[6] Choi Y, Gehring M, Johnson L, Hannon M, Harada JJ, Goldberg RB, Jacobsen SE, Fischer RL: DEMETER, a DNA glycosylase domain protein, is required for endosperm gene imprinting and seed viability in Arabidopsis. Cell 2002, 110(1):33–42. 12150995 10.1016/s0092-8674(02)00807-3

[7] Gehring M, Huh JH, Hsieh TF, Penterman J, Choi Y, Harada JJ, Goldberg RB, Fischer RL: DEMETER polycomb DNA glycosylase establishes MEDEA specific gene self-imprinting by allele-demethylation Cell 2006, 124(3):495–506. 16469697 10.1016/j.cell.2005.12.034 PMC4106368

[8] Ibarra CA, Feng X, Schoft VK, Hsieh TF, Uzawa R, Rodrigues JA, Zemach A, Chumak N, Machlicova A, Nishimura T : Active DNA demethylation in plant companion cells reinforces transposon methylation in gametes. Science 2012, 337(6100):1360–1364. 22984074 10.1126/science.1224839 PMC4034762

[9] Frost JM, Kim MY, Park GT, Hsieh PH, Nakamura M, Lin SJH, Yoo H, Choi J, Ikeda Y, Kinoshita T : FACT complex is required for DNA demethylation at heterochromatin during reproduction in Arabidopsis. Proc Natl Acad Sci U S A 2018, 115(20):E4720–E4729. 29712855 10.1073/pnas.1713333115 PMC5960277

[10] Park JS, Frost JM, Park K, Ohr H, Park GT, Kim S, Eom H, Lee I, Brooks JS, Fischer RL : Control of DEMETER DNA demethylase gene transcription in male and female gamete companion cells in Arabidopsis thaliana. Proc Natl Acad Sci U S A 2017, 114(8):2078–2083. 28130550 10.1073/pnas.1620592114 PMC5338364

[11] Hsieh TF, Ibarra CA, Silva P, Zemach A, Eshed-Williams L, Fischer RL, Zilberman D: Genome-wide demethylation of Arabidopsis endosperm. Science 2009, 324(5933):1451–1454. 19520962 10.1126/science.1172417 PMC4044190

[12] Ikeda Y, Kinoshita Y, Susaki D, Ikeda Y, Iwano M, Takayama S, Higashiyama T, Kakutani T, Kinoshita T: HMG domain containing SSRP1 is required for DNA demethylation and genomic imprinting in Arabidopsis. Dev Cell 2011, 21(3):589–596. 21920319 10.1016/j.devcel.2011.08.013

[13] Piquet S, Le Parc F, Bai SK, Chevallier O, Adam S, Polo SE: The Histone Chaperone FACT Coordinates H2A.X-Dependent Signaling and Repair of DNA Damage. Mol Cell 2018, 72(5):888–901 e887. 30344095 10.1016/j.molcel.2018.09.010 PMC6292839

[14] Belotserkovskaya R, Oh S, Bondarenko VA, Orphanides G, Studitsky VM, Reinberg D: FACT facilitates transcription-dependent nucleosome alteration. Science 2003, 301(5636):1090–1093. 12934006 10.1126/science.1085703

[15] Formosa T: The role of FACT in making and breaking nucleosomes. Biochim Biophys Acta 2012, 1819(3–4):247–255. 21807128 10.1016/j.bbagrm.2011.07.009 PMC3229669

[16] Heo K, Kim H, Choi SH, Choi J, Kim K, Gu J, Lieber MR, Yang AS, An W: FACT-mediated exchange of histone variant H2AX regulated by phosphorylation of H2AX and ADP-ribosylation of Spt16. Mol Cell 2008, 30(1):86–97. 18406329 10.1016/j.molcel.2008.02.029

[17] Dantuma NP, van Attikum H: Spatiotemporal regulation of posttranslational modifications in the DNA damage response. EMBO J 2016, 35(1):6–23. 26628622 10.15252/embj.201592595 PMC4717999

[18] Du LL, Nakamura TM, Russell P: Histone modification-dependent and -independent pathways for recruitment of checkpoint protein Crb2 to double-strand breaks. Genes Dev 2006, 20(12):1583–1596. 16778077 10.1101/gad.1422606 PMC1482479

[19] Sczepanski JT, Wong RS, McKnight JN, Bowman GD, Greenberg MM: Rapid DNA-protein cross-linking and strand scission by an abasic site in a nucleosome core particle. Proc Natl Acad Sci U S A 2010, 107(52):22475–22480. 21149689 10.1073/pnas.1012860108 PMC3012510

[20] Kotogany E, Dudits D, Horvath GV, Ayaydin F: A rapid and robust assay for detection of S-phase cell cycle progression in plant cells and tissues by using ethynyl deoxyuridine. Plant Methods 2010, 6(1):5. 20181034 10.1186/1746-4811-6-5 PMC2828981

[21] Culligan KM, Robertson CE, Foreman J, Doerner P, Britt AB: ATR and ATM play both distinct and additive roles in response to ionizing radiation. Plant J 2006, 48(6):947–961. 17227549 10.1111/j.1365-313X.2006.02931.x

[22] Lorkovic ZJ, Park C, Goiser M, Jiang D, Kurzbauer MT, Schlogelhofer P, Berger F: Compartmentalization of DNA Damage Response between Heterochromatin and Euchromatin Is Mediated by Distinct H2A Histone Variants. Current Biology 2017, 27(8):1192–1199. 28392109 10.1016/j.cub.2017.03.002

[23] Schoft VK, Chumak N, Choi Y, Hannon M, Garcia-Aguilar M, Machlicova A, Slusarz L, Mosiolek M, Park JS, Park GT : Function of the DEMETER DNA glycosylase in the Arabidopsis thaliana male gametophyte. Proc Natl Acad Sci U S A 2011, 108(19):8042–8047. 21518889 10.1073/pnas.1105117108 PMC3093457

[24] Park K, Kim MY, Vickers M, Park JS, Hyun Y, Okamoto T, Zilberman D, Fischer RL, Feng X, Choi Y : DNA demethylation is initiated in the central cells of Arabidopsis and rice. Proc Natl Acad Sci U S A 2016, 113(52):15138–15143. 27956642 10.1073/pnas.1619047114 PMC5206524

[25] Papareddy RK, Paldi K, Paulraj S, Kao P, Lutzmayer S, Nodine MD: Chromatin regulates expression of small RNAs to help maintain transposon methylome homeostasis in Arabidopsis. Genome Biol 2020, 21(1):251. 32943088 10.1186/s13059-020-02163-4 PMC7499886

[26] Sequeira-Mendes J, Araguez I, Peiro R, Mendez-Giraldez R, Zhang X, Jacobsen SE, Bastolla U, Gutierrez C: The Functional Topography of the Arabidopsis Genome Is Organized in a Reduced Number of Linear Motifs of Chromatin States. Plant Cell 2014, 26(6):2351–2366. 24934173 10.1105/tpc.114.124578 PMC4114938

[27] Celeste A, Difilippantonio S, Difilippantonio MJ, Fernandez-Capetillo O, Pilch DR, Sedelnikova OA, Eckhaus M, Ried T, Bonner WM, Nussenzweig A: H2AX haploinsufficiency modifies genomic stability and tumor susceptibility. Cell 2003, 114(3):371–383. 12914701 10.1016/s0092-8674(03)00567-1 PMC4737479

[28] Celeste A, Fernandez-Capetillo O, Kruhlak MJ, Pilch DR, Staudt DW, Lee A, Bonner RF, Bonner WM, Nussenzweig A: Histone H2AX phosphorylation is dispensable for the initial recognition of DNA breaks. Nat Cell Biol 2003, 5(7):675–679. 12792649 10.1038/ncb1004

[29] Rogakou EP, Sekeri-Pataryas KE: Histone variants of H2A and H3 families are regulated during in vitro aging in the same manner as during differentiation. Exp Gerontol 1999, 34(6):741–754. 10579635 10.1016/s0531-5565(99)00046-7

[30] Seo J, Kim SC, Lee HS, Kim JK, Shon HJ, Salleh NL, Desai KV, Lee JH, Kang ES, Kim JS : Genome-wide profiles of H2AX and gamma-H2AX differentiate endogenous and exogenous DNA damage hotspots in human cells. Nucleic Acids Res 2012, 40(13):5965–5974. 22467212 10.1093/nar/gks287 PMC3401470

[31] Yi H, Sardesai N, Fujinuma T, Chan CW, Veena, Gelvin SB: Constitutive expression exposes functional redundancy between the Arabidopsis histone H2A gene HTA1 and other H2A gene family members. Plant Cell 2006, 18(7):1575–1589. 16751347 10.1105/tpc.105.039719 PMC1488917

[32] Deal RB, Kandasamy MK, McKinney EC, Meagher RB: The nuclear actin-related protein ARP6 is a pleiotropic developmental regulator required for the maintenance of FLOWERING LOCUS C expression and repression of flowering in Arabidopsis. Plant Cell 2005, 17(10):2633–2646. 16141450 10.1105/tpc.105.035196 PMC1242262

[33] Smith AP, Jain A, Deal RB, Nagarajan VK, Poling MD, Raghothama KG, Meagher RB: Histone H2A.Z regulates the expression of several classes of phosphate starvation response genes but not as a transcriptional activator. Plant Physiol 2010, 152(1):217–225. 19897606 10.1104/pp.109.145532 PMC2799358

[34] Won SK, Lee YJ, Lee HY, Heo YK, Cho M, Cho HT: Cis-element- and transcriptome-based screening of root hair-specific genes and their functional characterization in Arabidopsis. Plant Physiol 2009, 150(3):1459–1473. 19448035 10.1104/pp.109.140905 PMC2705046

[35] Hwang Y, Choi HS, Cho HM, Cho HT: Tracheophytes Contain Conserved Orthologs of a Basic Helix-Loop-Helix Transcription Factor That Modulate ROOT HAIR SPECIFIC Genes. Plant Cell 2017, 29(1):39–53. 28087829 10.1105/tpc.16.00732 PMC5304353

[36] Mangano S, Denita-Juarez SP, Choi HS, Marzol E, Hwang Y, Ranocha P, Velasquez SM, Borassi C, Barberini ML, Aptekmann AA : Molecular link between auxin and ROS-mediated polar growth. Proc Natl Acad Sci U S A 2017, 114(20):5289–5294. 28461488 10.1073/pnas.1701536114 PMC5441824

[37] Yadav K, Garoff L, Huseby DL, Hughes D: Phenotypic and genetic barriers to establishment of horizontally transferred genes encoding ribosomal protection proteins. J Antimicrob Chemother 2021. 10.1093/jac/dkab056 PMC8120329 33655294

[38] Baroux C, Pecinka A, Fuchs J, Schubert I, Grossniklaus U: The triploid endosperm genome of Arabidopsis adopts a peculiar, parental-dosage-dependent chromatin organization. Plant Cell 2007, 19(6):1782–1794. 17557811 10.1105/tpc.106.046235 PMC1955730

[39] Waterworth WM, Wilson M, Wang D, Nuhse T, Warward S, Selley J, West CE: Phosphoproteomic analysis reveals plant DNA damage signalling pathways with a functional role for histone H2AX phosphorylation in plant growth under genotoxic stress. Plant J 2019, 100(5):1007–1021. 31410901 10.1111/tpj.14495 PMC6900162

[40] Lee SH, Cho HT: PINOID positively regulates auxin efflux in Arabidopsis root hair cells and tobacco cells. Plant Cell 2006, 18(7):1604–1616. 16731587 10.1105/tpc.105.035972 PMC1488908

[41] Kim S, Park JS, Lee J, Lee KK, Park OS, Choi HS, Seo PJ, Cho HT, Frost JM, Fischer RL : The DME demethylase regulates sporophyte gene expression, cell proliferation, differentiation, and meristem resurrection. Proc Natl Acad Sci U S A 2021, 118(29). 10.1073/pnas.2026806118 PMC8307533 34266952

[42] Min Y, Frost JM, Choi Y: Nuclear Chaperone ASF1 is Required for Gametogenesis in Arabidopsis thaliana. Scientific Reports 2019, 9(1):13959. 31562367 10.1038/s41598-019-50450-3 PMC6764951

